# Targeted Methylation Profiling of Single Laser-Capture Microdissected Post-Mortem Brain Cells by Adapted Limiting Dilution Bisulfite Pyrosequencing (LDBSP)

**DOI:** 10.3390/ijms232415571

**Published:** 2022-12-08

**Authors:** Renzo J. M. Riemens, Gunter Kenis, Jennifer Nolz, Sonia C. Susano Chaves, Diane Duroux, Ehsan Pishva, Diego Mastroeni, Kristel Van Steen, Thomas Haaf, Daniël L. A. van den Hove

**Affiliations:** 1 Department of Psychiatry and Neuropsychology, School for Mental Health and Neuroscience (MHeNs), Maastricht University, 6229 ER Maastricht, The Netherlands; 2 Institute of Human Genetics, Julius Maximilians University, 97074 Wuerzburg, Germany; 3 Biodesign Institute, Neurodegenerative Disease Research Center, Arizona State University, Tempe, AZ 85281, USA; 4 BIO3 Laboratory for Systems Genetics, GIGA-R Medical Genomics, University of Liège, 4000 Liège, Belgium; 5 BIO3 Laboratory for Systems Medicine, Department of Human Genetics, KU Leuven, 3000 Leuven, Belgium; 6 Laboratory of Translational Neuroscience, Department of Psychiatry, Psychosomatics and Psychotherapy, University of Wuerzburg, 97080 Wuerzburg, Germany

**Keywords:** limiting dilution bisulfite pyrosequencing, laser-capture microdissection, DNA methylation, single-cell, epigenetics

## Abstract

A reoccurring issue in neuroepigenomic studies, especially in the context of neurodegenerative disease, is the use of (heterogeneous) bulk tissue, which generates noise during epigenetic profiling. A workable solution to this issue is to quantify epigenetic patterns in individually isolated neuronal cells using laser capture microdissection (LCM). For this purpose, we established a novel approach for targeted DNA methylation profiling of individual genes that relies on a combination of LCM and limiting dilution bisulfite pyrosequencing (LDBSP). Using this approach, we determined cytosine-phosphate-guanine (CpG) methylation rates of single alleles derived from 50 neurons that were isolated from unfixed post-mortem brain tissue. In the present manuscript, we describe the general workflow and, as a showcase, demonstrate how targeted methylation analysis of various genes, in this case, *RHBDF2*, *OXT*, *TNXB*, *DNAJB13*, *PGLYRP1*, *C3*, and *LMX1B*, can be performed simultaneously. By doing so, we describe an adapted data analysis pipeline for LDBSP, allowing one to include and correct CpG methylation rates derived from multi-allele reactions. In addition, we show that the efficiency of LDBSP on DNA derived from LCM neurons is similar to the efficiency obtained in previously published studies using this technique on other cell types. Overall, the method described here provides the user with a more accurate estimation of the DNA methylation status of each target gene in the analyzed cell pools, thereby adding further validity to this approach.

## 1. Introduction

An increasing number of studies have implicated a central role for epigenetic mechanisms such as DNA methylation in the pathophysiology of neurodegenerative disorders, including Alzheimer’s disease (AD) [1,2]. However, the field of neuroepigenomics still faces many challenges that impede attempts to disentangle the exact contribution of DNA methylation alterations in the development and course of disorders like AD. A central issue is the cellular heterogeneity of the studied bulk tissue samples, which represents a significant source of noise in epigenetic profiling [3]. Next to drastically decreasing the signal-to-noise ratio, the use of bulk tissue samples does not allow one to conclude whether differences found between, e.g., patient and control brains are related to disease status rather than changes in cell-type composition [1]. Neurodegenerative diseases like AD are characterized by neuronal loss, concomitant with alterations in the brain cellular architecture [2]. Thus, differences in cellular composition between tissue samples from patients and control cases can lead to misinterpretation of the acquired epigenetic data. In addition, cell-type composition differences associated with tissue sampling itself may also introduce bias. Moreover, even in the case of comparable cellular proportions, heterogeneous tissues could still mask cell-type-specific modifications related to the disease, as changes in one cell type could dilute or oppose changes in another, thereby obscuring important cell subtype-specific aberrations when analyzed together [4].

To overcome the issue of cellular heterogeneity in methylome-wide studies, it is possible to correct for cell-type composition using advanced bioinformatics approaches [5,6,7]. However, there is an ongoing dispute concerning the validity of this approach [3,8,9]. A more accurate alternative, and the only reliable option in the case of targeted candidate gene-based approaches, is to profile epigenetic patterns in individually isolated cells that were selected, e.g., based on a cell type-specific marker. This latter approach does not only avoid noise that is induced by differences in the cell-type composition of the studied bulk tissue samples, but it also provides a more detailed overview of epigenetic profiles in individual cell types. In recent years, limiting dilution bisulfite pyrosequencing (LDBSP) has emerged as a cost-effective approach, allowing targeted bisulfite methylation profiling at a single cytosine-phosphate-guanine (CpG) site resolution in a single or a few cells [10]. The principle of this technique relies on an extensive dilution of the bisulfite-treated target DNA obtained from a single or a few cell(s) so that a maximum of one allele is present in each of the downstream reactions. Subsequently, each DNA molecule is separately amplified by (semi-) nested polymerase chain reactions (PCRs) and analyzed using pyrosequencing. Thus, when applied in conjunction with a highly selective isolation procedure, such as laser-capture microdissection (LCM) based on the immunoreactivity of a cell-specific marker, this technique offers an appealing approach for the detection of methylation patterns in individual cellular populations of the brain.

**Figure 1:**
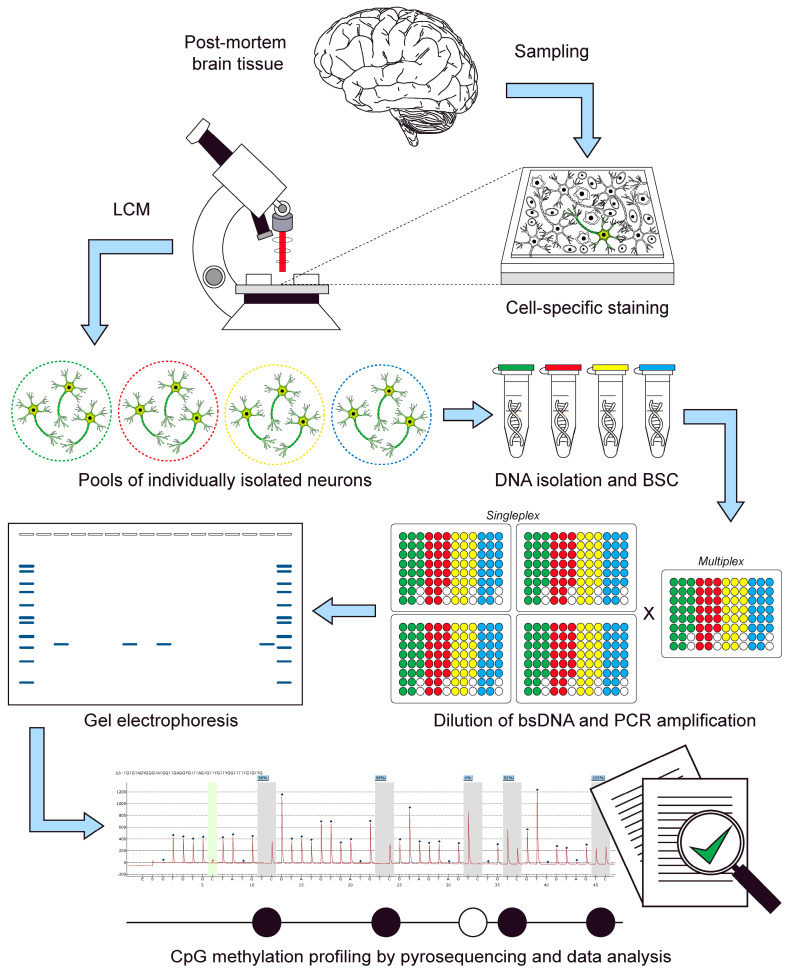
Overview of the limiting dilution bisulfite pyrosequencing (LDBPS) procedure for pools of individually isolated neurons using laser capture microdissection (LCM). Tissue from post-mortem brains is sampled and stained using a cell type-specific marker. Neurons are then isolated and divided into small cell pools. DNA derived from these neurons then undergoes a bisulfite conversion (BSC) treatment and is subsequently diluted to a single allele level. The bisulfite converted DNA (bsDNA) is then amplified twice by means of a (semi-)nested polymerase chain reaction (PCR) in multiplex-singleplex formation. Product-yielding reactions are then visualized on an agarose gel and the cytosine-phosphate-guanine (CpG) methylation status of these products is then profiled by means of pyrosequencing.

Although LDBSP has been shown to be compatible for individual 2–16 cell embryos, single germinal vesicle oocytes, and haploid sperm cells [10,11], to date, this technique has not been applied on ex vivo brain cells. In the present study, we, therefore, demonstrate, for the first time, that LDBSP can be successfully applied on pools of 50 neurons isolated by LCM from unfixed post-mortem brain tissue (Figure 1). Here, we describe the general adapted LCM-LDBSP pipeline and, as a showcase, demonstrate how a targeted methylation analysis can be performed in multiple genes, i.e., *RHBDF2*, *OXT*, *TNXB*, *DNAJB13*, *PGLYRP1*, *C3*, and *LMX1B*, simultaneously.

Importantly, diluting a DNA sample derived from 50 neurons to a single-allele-level will occasionally render reactions with more than one allele, a scenario that does not regularly occur when conducting LDBSP using one or just a few cells. Therefore, we describe an adapted data analysis pipeline designed explicitly for assessing pools of LCM neurons that allows to include and correct the methylation data for multi-allele reactions. This novel approach, which compromises a CpG-site-dependent calling procedure, combined with an integrated in-depth analysis of the raw CpG methylation rates, aims at avoiding unintentionally induced bias due to the blunt exclusion of reactions that likely contain more than one allele. Overall, the method described here allows the user to more accurately estimate average DNA methylation levels of target genes in the analyzed samples, thereby adding further validity to the experimental data acquired. In addition, based on the number of recovered alleles, we show that the efficiency of LDBSP on 50 neurons isolated with LCM from post-mortem brain tissues (4.90% on average per gene) is similar to the efficiency achieved in previously published studies (0.3% up to 25%) using this technique on other isolated cell types, including cell pools consisting of 10 oocytes, two-cell embryos, and 16-cell embryos [11,12,13].

## 2. Results

### 2.1. LDBSP Allows for Methylation Profiling of Pools of 50 LCM-Collected Neurons

To assess the compatibility of LDBSP on neurons that were individually isolated from unfixed post-mortem brain tissue using LCM, batches consisting of 1, 5, 10, and 50 cells were initially processed following the working procedures described in the present manuscript. Following LDBSP, only pools of 50 cells yielded a favorable amount of sequenceable PCR products for each gene. Next, 150 neurons per individual from 24 donors were isolated, divided over three pools of 50 cells each, and processed for further analysis. To ensure adequate dilution to a single-allele level, the dilution factor that is applied when conducting LDBSP should be larger than the maximum number of DNA molecules in the starting sample. For pragmatic reasons, at least half of the reactions should therefore not contain a PCR product after the procedure [11]. In our hands, on average, 4.29 PCR products were obtained per gene per pool of 50 neurons, hence a dilution factor of 22 was used. Of note, we did not observe any striking differences in the number of acquired products when increasing the number of dilutions (up to 96x). Overall, for *RHBDF2*, *OXT*, *TNXB*, *DNAJB13*, *PGLYRP1*, *C3*, and *LMX1B*, 315, 241, 237, 416, 233, 189, and 532 PCR products, respectively, were obtained (Table 1). All of these were then successfully taken forward for methylation profiling using pyrosequencing.

A summary of all the LDBSP parameters for pools of 50 neurons isolated from unfixed post-mortem brain tissue using laser capture microdissection (LCM). Displayed are the total number of obtained singleplex polymerase chain reaction (PCR) products for each gene, the total amount of included and excluded products according to the traditional LDBSP method for the downstream data analysis, as well as the total amount of estimated alleles according to the novel LDBSP data analysis approach. Please refer to the written text for further specifications on both allele estimation methods and criteria.

### 2.2. LDBSP on Pools of 50 Neurons Occasionally Renders Reactions with More than One Target Allele

As the principle of LDBSP is based on the seclusion of individual alleles, the vast majority of PCR products should theoretically represent an amplicon derived from one DNA molecule [10]. As a given CpG site on a single allele is simply methylated or not, pyrosequencing of these PCR products should result in obtaining binary CpG methylation readouts, i.e., the percentage of methylation should approach 0% for unmethylated and 100% for methylated CpG sites. Because the quantitative measurement of a CpG site by pyrosequencing does not only depend on its methylation status, but also on the sensitivity of the assay and other technological factors, earlier studies have considered CpG methylation values of <20% and >80% indicative of unmethylated and methylated CpG sites, respectively [10,11,12]. Moreover, in those same studies, products that contained at least one CpG methylation value between 20% and 80% were excluded from further downstream analyses, as these were considered to represent measures of multiple target alleles in a single reaction or from other technological artifacts. Importantly, as previous studies have mainly applied LDBSP on a single or a few cells, the occurrence of multi-allele reactions has often been negligible [11].

When applying LDBSP on pools containing a larger number of cells, i.e., 50 neurons in our case, one can observe that a substantial proportion of the obtained products do not meet the traditional LDBSP inclusion criteria. Evidently, processing DNA isolated from a pool of 50 neurons increases the chance of allele clumping when compared to conducting LDBSP on a single or just a few cells. As such, when two different alleles with an opposite methylation status for at least one CpG site end up in the same reaction, the methylation value obtained for specifically this site should approach ~50%. Along similar lines, when three alleles end up in the same reaction with a different methylation status for at least one CpG site, then the methylation value obtained for specifically this site should approach either ~33.33% or ~66.66%. From all the PCR products of *RHBDF2*, *OXT*, *TNXB*, *DNAJB13*, *PGLYRP1*, *C3*, and *LMX1B*, respectively, 5.40%, 8.71%, 15.19%, 16.83%, 12.45%, 1.59%, and 10.90%, would have been omitted from further downstream analyses, as at least one CpG site in these products demonstrated a value between 20% and 80% (Table 1). A thorough inspection of these deviating CpG methylation values revealed that the vast majority approached either 50% or 33%/67%, indicative of the presence of two or three alleles. Thus, based on these typical patterns of CpG methylation values, we concluded that a considerable amount of the LDBSP data could be attributed to reactions that contained more than one target allele, hence suggesting an adapted protocol for estimating methylation rates using this approach is required. Evidently, one can also obtain methylation values of 0% or 100% in the case of two or three allele reactions, which demands a reliable determination of the number of alleles in each reaction.

### 2.3. An Integrated Analysis of CpG Read-Outs for LDBSP Data with Correction for Multi-Allele Reactions

To correct for the number of estimated alleles present in each of the PCRs, we established a novel method for the downstream data analysis. For this purpose, thresholds of (1) ≤8.33% and ≥91.33%, (2) 50 ± 8.33%, and (3) 33.33 ± 8.33% and 66.66 ± 8.33%, were set for the CpG methylation values, enabling assessing both 1-, 2- and 3-allele reactions, respectively. Accordingly, individual CpG sites that fell within the first, second, and third threshold range were called and considered indicative of the potential presence of one, two, and three alleles, respectively. A definitive (total) allele score for each product was then assigned following a multi-step filtering process that was based on the criteria described hereafter. All products solely displaying binary CpG methylation patterns, i.e., when every CpG site within an amplicon displays methylation levels within the first threshold range, were directly considered to be derived from single allele reactions, as the CpG methylation profiles for these products displayed a typical binary pattern that is expected for a single DNA molecule. Similarly, and based on the assumption described in the previous section, all products displaying CpG methylation values that fell only in the first and second, but not third, or only in the first and third, but not second, threshold ranges were scored as two or three alleles, respectively. 

Subsequently, products containing CpG methylation values that fell outside of the assigned threshold ranges, i.e., between 8.33–25% and 75–91.33%, and/or products with values that were indicative of both two and three alleles simultaneously, were assessed. The observation of such more ambiguous products displaying small deviations from the assigned threshold values is likely to be caused by technical variation induced during the PCR and pyrosequencing procedure and the more stringent thresholds used in the present study in this respect (8.33% versus 20% in previous studies; see above). All of these products were therefore thoroughly inspected by two investigators that were blinded to the experimental conditions, and a decision on the total number of alleles present in each reaction, i.e., one, two, or three alleles, was made independently while taking into account a combination of different factors. These included but were not limited to, small technological variation that was previously observed during sensitivity testing of the assays, the directionality and methylation status of other CpG sites in the same product, and the total number of dominant allele indicators, i.e., whether a product demonstrated more or less CpG sites suggestive for either two or three alleles. Furthermore, a likelihood estimation for each CpG site was made by taking into account the methylation status frequency on other gene-specific products obtained from the same individual, as well as from identical products obtained from other individuals. A cross-comparison between the independent score sheets was then performed (99.46% overlap per gene on average) and reactions with a deviating score between the first two investigators were assessed by a third (blinded) investigator. A final allele number was then assigned for these reactions based on the overlap between the score sheets of the third and first two investigators, i.e., when two out of the three investigators assigned the same score, this allele number was used for the respective reaction.

**Figure 2:**
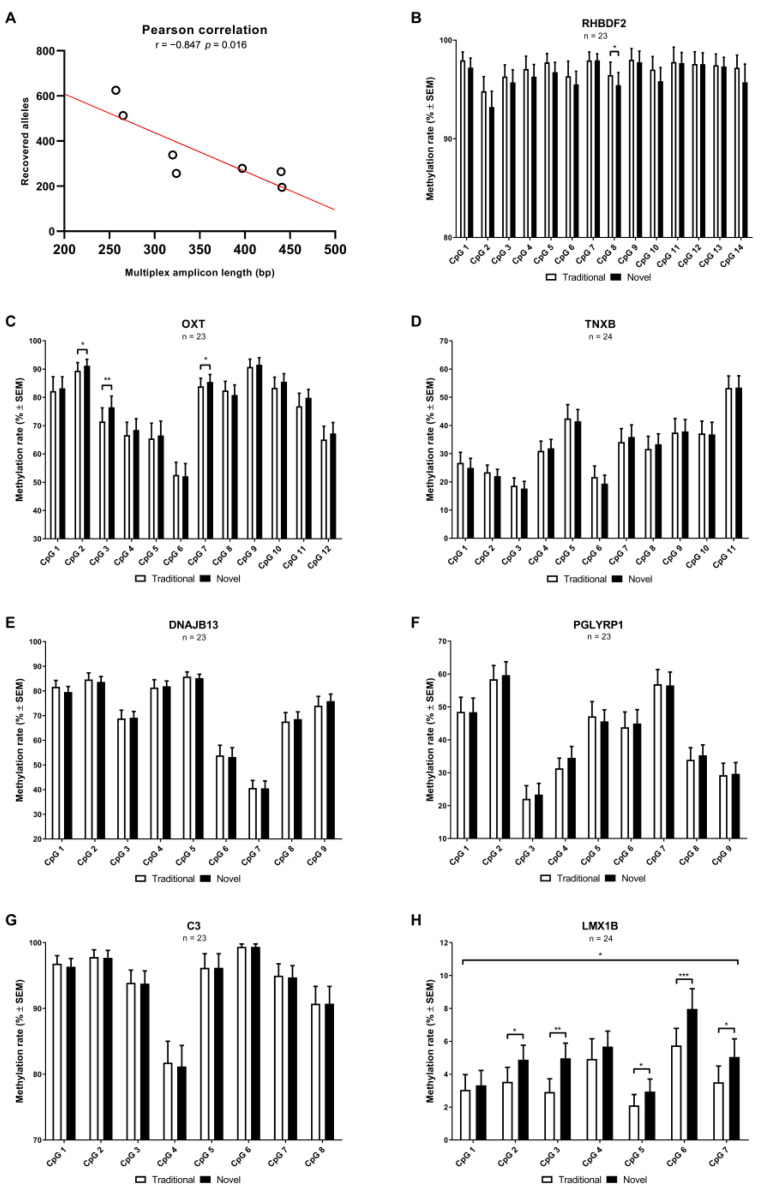
(**A**) Displayed is the Pearson correlation test was performed to assess whether there was a correlation between the number of recovered alleles and multiplex amplicon length of each gene. (**B**–**H**) Shown for each target gene are the paired cytosine-phosphate-guanine (CpG) methylation rates (mean percentage ± standard error of the mean [SEM]) estimated by both the traditional and novel downstream data analysis for limiting dilution bisulfite pyrosequencing (LDBSP) on pools of 50 neurons. A one-way repeated measures multivariate analysis of variance (MANOVA) was performed in order to determine whether there was a combined significant difference in the CpG methylation rates estimated by both methods. Subsequently, a univariate analysis was performed in order to identify differences between the methylation rates for each individual CpG site. A *p*-value of <0.05 was considered statistically significant. Significant findings for the multivariate and univariate tests are indicated with an asterix (* = *p* < 0.05; ** = *p* < 0.01; *** = *p* < 0.001).

Overall, we estimated that from the obtained PCR products for all target genes, 91.26% were derived from single alleles, whereas 3.28% were derived from two alleles, and 5.46% were derived from three alleles (Table 1 and Figure S1). By taking into account these multi-allele reactions, we, therefore, estimated that on average 352.86 alleles were recovered per target gene, with an average recovery rate of 4.90%. It has previously been demonstrated that shorter assays generally have a higher recovery rate for LDBSP [11], something that could also be observed in our dataset. In fact, the highest number of alleles were recovered for the shortest assay (*LMX1B*, 625 alleles; multiplex, 257 base pairs (bp); singleplex, 249 bp), while the lowest number was recovered for the longest assay (*C3*, 195 alleles; multiplex 441 bp; singleplex, 364 bp). Accordingly, a Pearson correlation test revealed a strong negative correlation (r = −0.847, *p* = 0.016) between the number of recovered alleles and multiplex amplicon length, thereby confirming previous observations (Figure 2A).

Based on the final allele estimations above, the CpG methylation rates, representing the percentage of methylated CpG sites from the total number of recovered alleles per gene, were calculated per individual. An overview of the average CpG methylation rates can be found in Figure 2B–H for each target gene. In addition to estimating these rates by the novel method described above, we also quantified them according to the traditional LDBSP approach that is based on excluding multi-allele reactions.

### 2.4. Gene-Specific Changes in the CpG Methylation Data Based on the Novel Integrative Analysis

Next, for the CpG site methylation rates of each gene, a one-way repeated measures MANOVA was performed to determine whether there was a combined significant difference in the CpG methylation rates estimated by the traditional and novel LDBSP data analysis approach described above (Table 2). A significant effect (*p* = 0.017) was identified for *LMX1B*, demonstrating that, for this gene, the estimated CpG methylation rates are different depending on the applied calling procedure. A subsequent univariate analysis for each individual CpG site, indicated that the estimated methylation rates for 5 out of 7 sites were significantly different between the two approaches (CpG 2, *p* = 0.012; CpG 3, *p* = 0.010; CpG 5, *p* = 0.040; CpG 6, *p* = 0.001; CpG 7, *p* = 0.008). Whereas no significant combined effect was identified for the other target genes, the univariate analyses did reveal that the estimated methylation rates for one CpG site in *RHBDF2* and three CpG sites in *OXT* were different between the traditional and novel methods. While for *RHBDF2* CpG site 8 (*p* = 0.047) was significantly different, CpG site 2 (*p* = 0.048), 3 (*p* = 0.005) and 7 (*p* = 0.033) of *OXT* differed between the two approaches. Altogether, these findings demonstrate that the obtained methylation rates can be significantly affected by the method that is applied for downstream data analysis when conducting LDBSP on pools of 50 neurons. In other words, when excluding reactions that likely contain multiple alleles, instead of correcting the derived methylation rates based on the number of estimated alleles present in the reaction, the eventual LDBSP data can differ significantly. For this reason, and to prevent potential bias in the experimental outcomes, we strongly suggest applying this adapted approach for LDBSP when analyzing pools including larger numbers of cells. Overall, this novel pipeline approach provides a closer estimate of the true CpG methylation rates for a target gene.

## 3. Discussion

In the present study, we demonstrated, for the first time, that LDBSP can be successfully applied on LCM neurons derived from unfixed post-mortem brains. In brief, brain tissue sections were stained for a neuronal subtype-specific marker, i.e., 5-HT. Immuno-positive cells were then individually isolated using LCM and subsequently divided into small pools of 50 neurons. Next, the bisulfite-converted DNA isolated from a pool of neurons was diluted to a single-allele-level and then amplified using (semi-)nested PCRs in multiplex-singleplex formation, targeting *RHBDF2*, *OXT*, *TNXB*, *DNAJB13*, *PGLYRP1*, *C3*, and *LMX1B*, simultaneously. Finally, the methylation status of the target genes was then quantified using bisulfite pyrosequencing. 

In contrast to what most previous studies reported [10,11,12,13,14,15], LDBSP on pools of 50 neurons renders a considerable degree (8.74% on average per gene) of the downstream PCRs containing more than one target allele. The presence of these multiple alleles could be identified by the observed ‘aberrant’ CpG methylation values obtained from the respective reactions. Pyrosequencing of single alleles normally results in the derivation of a binary CpG methylation readout, that is the methylation values approach 0% for unmethylated and 100% for methylated sites [10]. Although a binary CpG methylation pattern could be detected in the vast majority of reactions (91.26%), for 3.28% and 5.46% of the sequenced products, we obtained values that approached 50% or 33/67% for at least one CpG site, respectively. As such, we discovered that these reactions contained either two or three alleles, in which at least one DNA molecule had an opposite CpG methylation status as compared to the other allele(s) present in the same reaction. Traditionally, thresholds of <20% and >80% have been applied to define the methylation status of CpG sites, and all products displaying intermediate values (20–80%), suggesting the presence of more than one allele, were excluded from the downstream analysis [10,11,12,13,14,15]. However, we now argue that, particularly when the degree of these reactions is more substantial, exclusion might influence the data negatively, either by inducing bias or by reducing or reinforcing effect sizes. 

We therefore established a novel data analysis pipeline that allows one to include and correct the LDBSP data for these multi-allele reactions, hence providing a more accurate estimation of the CpG methylation rates. For this purpose, novel adjusted CpG site thresholds were applied to identify reactions that contained one, two or three alleles, respectively. Based on these thresholds, a stepwise CpG site calling procedure was performed in order to assign an allele score. For each gene, we then compared the CpG methylation rates estimated both by the traditional and our novel data analysis approach described above. Strikingly, significant differences in the combined CpG effects for *LMX1B*, as well as for individual CpG sites within *LMX1B*, *RHBDF2*, and *OXT* were identified. Thus, these findings emphasized that the derived LDBSP methylation data can significantly differ depending on the method that is being applied for the downstream data analysis. Importantly, this will particularly affect loci that for biological reasons display a varying methylation status over all the recovered alleles, that is when part of them is methylated and part of them unmethylated at one or more CpG sites. In such a scenario, the average methylation rates will likely fall in the intermediate range of 20–80% and, hence, the chances of obtaining multi-allele reactions with values outside of the traditional threshold will therefore be higher. As such, the probability of detecting differences between both methods is also dependent on the average methylation scores for the gene of interest. In either way, when LDBSP renders reactions with more than one target allele, independent of the cell types that are analyzed, correcting for the number of alleles provides a more accurate representation of the true methylation rates obtained for a specific target gene, hence adding more validity to the data.

One important consideration is that dependent on the design of an individual experiment, potentially more than three alleles might be present in an individual reaction. This would mean that the estimation of allele numbers in a single reaction based on CpG methylation values becomes too ambiguous, as one also needs to consider potential technical variation that might affect these read-outs. In our hands, the proportion of reactions that contain more than three alleles seemed to be very little or even negligible, and these reactions therefore hardly affect the estimated methylation data. Even when present, the method proposed in the present manuscript will most likely classify these reactions as three alleles and, hence, the methylation data obtained from these reactions has been partially corrected when compared to the traditional analysis pipeline. Evidently, when ending up with a high degree of (>3) multi-allele reactions, it is advised to further dilute the DNA.

Another important consideration is that LDBSP does not allow the user to detect reactions that appear to contain only one allele, which in reality contain two or more alleles with an identical methylation pattern. In such a scenario, the methylation profile of a PCR product would appear binary, hence suggesting the presence of only one target allele. Indeed, this represents an issue independent of the downstream data analysis used to analyze the CpG methylation rates, i.e., the traditional or novel approach proposed here. Overall, this becomes a bigger challenge when for biological reasons, the CpG methylation rates of a target gene approach either 0 or 100%, meaning that most of the recovered alleles will display either fully unmethylated or methylated CpG sites, respectively. In our study, this might therefore have affected genes such as *LMX1B*, *C3*, and *RHBDF2*. However, we often observed that at least one of the CpG sites in these gene-derived products displayed an opposite methylation status compared to the other sites derived from the same product. In other words, the methylation status of all CpG sites in a target gene was not always identical and often displayed a deviating methylation state for one site when compared to the others. For this reason, the occurrence of these opposite methylation patterns at single CpG sites still allowed us to detect the presence of multiple alleles when such molecules end up in a reaction with other alleles showing a more homogenous pattern of CpG methylation. It is therefore advisable to always target a substantial number of CpG sites per target gene, e.g., 7–14 in the present manuscript, to increase the chance of obtaining, and hence detecting, at least one site with a ‘non-binary’ methylation status for one of the target alleles. As such, a read-out of such a reaction would demonstrate an intermediate methylation value, e.g., ~50% and ~33% or ~67%, for specifically this CpG site. Therefore, when targeting only a few or even a single CpG site, further diluting the DNA may be advisory.

Undoubtedly, when conducting LDBSP, it is paramount to ensure proper dilution of the isolated target DNA a priori to minimize the occurrence of multi-allele reactions, even before one ought to correct the derived methylation values proposed in the present manuscript. LDBSP is thought to follow a Poisson distribution, meaning that with increasing dilution the chances of obtaining multi-allele reactions should become smaller. As a benchmark, it is therefore advised to use more dilutions than the number of target alleles present in the reactions, whilst taking into account potential loss due to handling. Adequate dilution to a single allele level can be assessed by the ratio between the number of product-yielding and non-product-yielding reactions, of which the latter should occur more often than the former. Although in the present study the number of product-yielding reactions was substantially lower (19.51% on average) compared to the downstream reactions that did not contain a product (80.49% on average), we still obtained reactions with more than one target allele, suggesting that other technological factors, or biochemical or biophysical properties of the DNA, play a role as well. Nevertheless, in a scenario where the DNA has been appropriately diluted and where multi-allele reactions are still encountered, it will likely remain more appropriate to correct methylation values for these reactions than excluding them from the data analysis. Of note, simply further diluting the DNA will be at the expense of throughput and comes along with additional costs.

Finally, to demonstrate the overall efficiency of the approach, we made a final estimation of the total number of alleles that were recovered after correcting for the number of alleles present in a single reaction. Assuming a 100% DNA recovery starting from three pools of 50 neurons, we estimated that the allele recovery rate of LDBSP on pools of 50 neurons isolated with LCM is 4.90% per gene of the total number of alleles. In comparison, previous studies have estimated recovery rates ranging from 0.3% up to 25% using intact cell pools consisting of 10 oocytes, two-cell embryos, and 16-cell embryos [11,12,13]. The recovery rate achieved in the present study on the pools of 50 neurons is therefore similar to other studies using these other cell types, even though a higher amount of starting material is needed when conducting the technique on neurons isolated with LCM. Nevertheless, one should not overlook the differences in sample collection procedures that were applied in these studies. In fact, the success of extracting DNA from cells isolated with LCM is dependent on several critical factors, e.g., cutting procedures, the thickness of the sections, staining procedures, exposure to the heat produced by the laser, and the DNA isolation process, which all could affect the eventual quantity (and quality) of DNA that is recovered from the cells. Furthermore, the degradation and low complexity of bisulfite-converted DNA oppose another challenge for the methylation analysis of small amounts of DNA from only a few cells. As such, a previous study demonstrated that shorter assays generally have a higher recovery rate for LDBSP [11]. Interestingly, also in our study the highest number of alleles were recovered for the shortest assay, while the lowest number was recovered for the longest assay. Accordingly, a strong negative correlation between the number of recovered alleles and multiplex amplicon length was identified, thereby confirming previous observations.

In conclusion, the approach described here, relying on a combination of LDBSP with LCM, offers a novel and alternative strategy to single-cell bisulfite sequencing techniques that can be applied for the study of DNA methylation marks in the human brain. Moreover, the approach offers a workable solution for the challenge of tissue and cell-type specificity as encountered in the field of neuroepigenomics. In fact, LDBSP on pools of neuronal populations allows one to determine DNA methylation profiles in a multi-targeted and cell subtype-specific manner, hence avoiding potential noise in epigenetic data that is induced by analyzing heterogeneous tissue samples. Aside from allowing the identification of methylation marks in individual neuronal cells, we expect that similar strategies using other isolation techniques and other cell subtypes in combination with LDBSP will be increasingly valuable for future neuroepigenomic studies.

## 4. Materials and Methods

A step-wise overview of the LDBSP protocol is available in Appendix A.

### 4.1. Ethics Statement

Written informed consent for brain autopsies was obtained in compliance with institutional guidelines of the Banner Sun Health Research Institute (BSHRI, Sun City, AZ, USA). The Banner Sun Health Research Institute Review Board approved the entire study, including the recruitment, enrollment, and autopsy procedures. Each individual and their respective relative(s) consented to a brain autopsy for scientific research as part of the BSHRI Brain and Body Donation Program (BBDP). The human brain tissue used in this study was derived from routine autopsies, fully qualifying for 4C exemption by the National Institute of Health (NIH) guidelines [16]. All samples were analyzed anonymously throughout the experimental procedures.

### 4.2. Sample Collection

Frozen unfixed dorsal raphe nucleus (DRN) tissue from 24 individuals (12 female AD and 12 female age-matched non-demented control cases) was collected at the BSHRI. Brain samples were frozen and stored at −80 °C after the autopsy, with an average post-mortem interval (PMI) of 2.69 ± 0.82 h. A final diagnosis of AD or non-demented healthy control was made based on the NIH AD Center criteria [16]. Comorbidity with any other type of dementia, mild cognitive impairment, cerebrovascular disorders, and the presence of non-microscopic infarcts were applied as exclusion criteria. For demographic and other relevant information about the studied samples, please refer to Table S1.

### 4.3. Immunohistochemistry

Frozen DRN tissue sections of 10 μm were mounted onto polyethylene naphthalate (PEN) slides and fixed in ice-cold 50% acetone/50% ethanol solution for 5 min on ice. Sections were washed in ice-cold phosphate-buffered saline (PBS), blocked in 1% hydrogen peroxide for 2 min, followed by three quick submersions in ice-cold PBS. Sections were then placed in a dilution of the primary antibody against serotonin (5-HT; Abcam, ab66047) in PBS for 10 min at room temperature. After the incubation, sections were washed three times in PBS and incubated with avidin-biotin complex in PBS for 10 min at room temperature. Next, sections were washed three times in 50 mM Tris buffer and immersed in 3.3′-diaminobenzidine (DAB) solution (9.3 mL 50 mM Tris; 200 μL DAB (5 mg/mL); 500 μL saturated nickel, and 4 μL of 1% hydrogen peroxidase) for 5 min, followed by two quick rinses in 50 mM Tris to stop the reaction. All sections were stored at −80 °C until further processing. 

### 4.4. Laser-Capture Microdissection

5-HT is a monoamine neurotransmitter that is specifically expressed by serotonergic neurons [17]. For this reason, LCM of serotonergic neurons from the DRN sections was performed based on 5-HT-immunoreactivity. In brief, sections were dipped in 100% ethanol, allowed to dry, and loaded onto a Leica AS-LMD LCM microscope (Leica, Wetzlar, Germany). Single serotonergic neurons were cut and then dropped into an inverted microcentrifuge cap containing 10 μL of Tris-EDTA (TE) buffer. Per individual, 150 serotonergic neurons were captured at 20× magnification and divided into small pools of 50 cells per microcentrifuge tube, i.e., yielding three pools of 50 neurons per subject. All isolated cells were stored at −80 °C until further processing.

### 4.5. DNA Isolation and Sodium Bisulfite Treatment

Genomic DNA from a pool of 50 neurons was isolated and bisulfite-converted using the EZ DNA Methylation-Direct Kit (Zymo Research, Irvine, CA, USA) with the following adjustments. In brief, 1 µL of proteinase K (20 µg/µL) and 11 µL of M-Digestion buffer (2X) were added to a microcentrifuge tube containing the cells and incubated overnight at 50 °C. Subsequently, the complete lysate was transferred to a PCR tube, and 143 µL of bisulfite conversion reagent was used to wash out the digestion tube before adding it to the sample. Bisulfite conversion was performed in a thermal cycler running at 98°C for 8 min and then at 64 °C for 3.5 h. A volume of 200 µL binding buffer was added to the spin column before loading the bisulfite-converted sample. The PCR tube used for bisulfite conversion was washed out twice by first adding 200 µL of binding buffer to the tube and then by transferring this volume to the sample-containing column. After centrifugation (10,000× *g*; 30 s), the column was washed with 100 µL washing buffer, incubated for 15 min with 200 µL desulfonation buffer, and washed twice again with 200 µL washing buffer. The bisulfite-converted DNA was eluted in a single Eppendorf tube by running 20 µL of elution buffer through the column twice (Two times at 10,000× *g*; 30 s). Eppendorf LoBind microcentrifuge tubes (Merck KGaA, Darmstadt, Germany) and TipOne Low Retention Tips (STARLAB, Hamburg, Germany) with low affinity for DNA were used throughout the whole procedure. Multiplex PCR amplifications were performed directly after elution of the bisulfite-converted DNA.

### 4.6. Multiplex Polymerase Chain Reaction

All assays were based on a (semi-)nested PCR design and amplified in multiplex-singleplex formation. Primers were designed with the PyroMark Assay Design 2.0 software (Qiagen, Hilden, Germany; see Table S2). Bisulfite-treated DNA derived from a pool of 50 neurons was diluted to a single allele level by adding a multiplex PCR mixture with a capacity of 22 individual reactions to the sample (determined empirically). Each multiplex PCR made use of 2.5 μL PCR buffer (10X) with 20 mM MgCl2, 0.5 μL 10 mM dNTP mix, 1 μL of each primer (10 μM stock) and 0.2 μL (5 U/μL) FastStart™ Taq DNA Polymerase (Roche Diagnostics GmbH, Mannheim, Germany) in a total volume of 25 μL. After adding the bisulfite DNA to the complete mixture, the sample was pipetted up and down to homogeneously disperse all bisulfite DNA molecules throughout the solution, and fractions of 25 µL were divided over 22 wells of a microtiter plate. Multiplex PCRs were then performed with an initial denaturation step at 95 °C for 5 min, followed by 43 cycles with denaturation at 95 °C for 30 s, annealing at 56 °C for 30 s and extension at 72 °C for 1 min, with a final extension step at 72 °C for 7 min.

### 4.7. Singleplex Polymerase Chain Reaction

For each singleplex PCR, 1 µL of the multiplex product was used as a template. In addition, every singleplex PCR made use of 2.5 μL PCR buffer (10X) with 20 mM MgCl2, 0.5 μL 10 mM dNTP mix, 1 μL of each primer (10 μM stock) and 0.2 μL (5 U/μL) FastStart™ Taq DNA Polymerase (Roche Diagnostics GmbH) in a total volume of 25 μL. Amplifications for each of the target genes were then performed with an initial denaturation step at 95 °C for 5 min, followed by 45 cycles with denaturation at 95 °C for 30 s, annealing at 58 °C for 30 s, and extension at 72 °C for 1 min, with a final extension step at 72 °C for 7 min. Reactions that yielded a singleplex PCR product were identified on an agarose gel and 3 μL of the product was utilized per assay for bisulfite pyrosequencing.

### 4.8. Bisulfite Pyrosequencing

The PyroMark Q96 MD pyrosequencing system (Qiagen) with the PyroMark Gold Q96 CDT reagent kit (Qiagen, Hilden, Germany) were used according to the manufacturer’s instructions. Methylation levels at single CpG resolution were quantified with the Pyro Q-CpG 1.0.9 software (Qiagen). All assays were tested for their sensitivity using the EpiTect PCR Control DNA Set (Qiagen). For further details on the pyrosequencing assays and sequencing primers, please refer to Table S2.

### 4.9. Statistical Analysis

All statistical analyses were performed with the IBM SPSS Statistics software version 25. To determine whether the estimated CpG methylation rates were significantly different between the novel and the traditional LDBSP data analysis pipeline, a one-way repeated measures multivariate analysis of variance (MANOVA) was performed for each target gene. Multivariate analysis was performed to evaluate whether there was a combined difference in CpG methylation rates measured over all CpG sites, followed by a univariate test for each site. A Pearson correlation test was performed to assess whether there was a correlation between the number of recovered alleles and multiplex amplicon length. For all statistical analyses, a *p*-value of <0.05 was considered statistically significant.

## Figures and Tables

**Table 1:** Overview of the limiting dilution bisulfite pyrosequencing (LDBSP) parameters.

*Singleplex PCR*	**RHBDF2**	**OXT**	**TNXB**	**DNAJB13**	**PGLYRP1**	**C3**	**LMX1B**	**Mean**
**Number (+ percentage) of reactions with PCR product**	315 (19.89)	241 (15.21)	237 (14.96)	416 (26.26)	233 (14.71)	189 (11.93)	532 (33.59)	309 (19.51)
*Allele estimation traditional LDBSP criteria*								
**Number (+ percentage) of included reactions (1 allele)**	298 (94.60)	220 (91.29)	201 (84.81)	346 (83.17)	204 (87.55)	186(98.41)	474 (89.10)	275.57 (89.18)
**Number (+ percentage) of excluded reactions (multi-allele/artifact)**	17 (5.40)	21 (8.71)	36 (15.19)	70 (16.83)	29 (12.45)	3 (1.59)	58 (10.90)	33.43 (10.82)
*Allele estimation novel LDBSP criteria*								
**Number (+ percentage) of reactions with 1 allele**	300 (95.24)	232 (96.27)	217 (91.56)	356 (85.58)	208 (89.27)	186 (98.41)	475 (89.29)	282 (91.26)
**Number (+ percentage) of reactions with 2 alleles**	7 (2.22)	3 (1.24)	13 (5.49)	23 (5.53)	4 (1.72)	0 (0.00)	21 (3.95)	10.14 (3.28)
**Number (+ percentage) of reactions with 3 alleles**	8 (2.54)	6 (2.49)	7 (2.95)	37 (8.89)	21 (9.01)	3 (1.59)	36 (6.77)	16.86 (5.46)
**Number (+ percentage) of multi-allele reactions**	15 (4.76)	9 (3.73)	20 (8.44)	60 (14.42)	25 (10.73)	3 (1.59)	57 (10.71)	27 (8.74)
**Number (+ percentage) of recovered alleles**	338 (4.69)	256 (3.56)	264 (3.67)	513 (7.13)	279 (3.88)	195 (2.71)	625 (8.68)	352.86 (4.90)

**Table 2:** One-way repeated measures multivariate analysis of variance (MANOVA).

*Multivariate test*	**RHBDF2**	**OXT**	**TNXB**	**DNAJB13**	**PGLYRP1**	**C3**	**LMX1B**
**Combined effect**	0.482	0.474	0.340	0.104	0.395	0.385	0.017 *
*Univariate tests*							
**CpG 1**	0.193	0.544	0.160	0.214	0.962	0.328	0.449
**CpG 2**	0.063	0.048 *	0.169	0.393	0.331	0.328	0.012 *
**CpG 3**	0.305	0.005 **	0.380	0.882	0.527	0.328	0.010 **
**CpG 4**	0.288	0.407	0.595	0.778	0.090	0.312	0.310
**CpG 5**	0.142	0.581	0.657	0.575	0.519	1.000	0.040 *
**CpG 6**	0.293	0.692	0.169	0.716	0.517	1.000	0.001 ***
**CpG 7**	0.973	0.033 *	0.212	0.922	0.863	0.328	0.008 **
**CpG 8**	0.047 *	0.349	0.450	0.600	0.544	1.000	-
**CpG 9**	0.328	0.381	0.868	0.203	0.857	-	-
**CpG 10**	0.053	0.087	0.728	-	-	-	-
**CpG 11**	0.859	0.228	0.910	-	-	-	-
**CpG 12**	0.963	0.394	-	-	-	-	-
**CpG 13**	0.814	-	-	-	-	-	-
**CpG 14**	0.083	-	-	-	-	-	-

Displayed are the *p*-values for both the multivariate test of the combined cytosine-phosphate-guanine (CpG) effects and the univariate tests for each individual CpG site per target gene. A *p*-value of <0.05 was considered as stastically significant. Significant findings for the multivariate and univariate tests are indicated with an asterix (* = *p* <0.05; ** = *p* <0.01; *** = *p* <0.001).

## Data Availability

All data associated with this study are available through the corresponding author upon request.

## Supplementary Materials

The following supporting information can be downloaded at: https://www.mdpi.com/article/10.3390/ijms232415571/s1.

## Appendix A

**Limiting dilution bisulfite pyrosequencing protocol for 50 laser capture microdissected cells**

***Reagents***

- Laser capture microdissected cells (pools of 50 cells) in 8 µL Tris-Ethylenediaminetetraacetic acid (TE) buffer or phosphate-buffered saline (PBS)
- FastStart™ Taq DNA Polymerase (Sigma Aldrich, cat. no. 12032929001)
- EZ DNA Methylation-Direct Kit (Zymo Research, cat. no. D5020)
- Agarose
- DNA loading dye, e.g., Orange G DNA Loading Dye (ThermoFisher Scientific, cat. no. R0631)
- Nucleic acid gel stain, e.g., GelsGelStarTM Nucleic Acid Gel Stain (Lonza, cat. no. 50535)
- DNA 100 bp ladder, e.g., 100 bp DNA Ladder (ThermoFisher Scientific, cat. no. 15628050)
- PyroMark Gold Q96 CDT reagent kit (Qiagen, cat. no. 972824)

***Equipment***

- Manual pipettes
- Multichannel pipette
- Pipette filtered tips
- Eppendorf LoBind microcentrifuge tubes (Merck KGaA, Darmstadt, Germany, cat. no. 022431021)
- TipOne Low Retention Tips (STARLAB, Hamburg, Germany, cat. no. S1180-8810)
- Eppendorf^®^ ThermoMixer^®^ F1.5 (Sigma Aldrich, cat. no. EP5384000012)
- Microcentrifuge with PCR tube adaptors
- Thermal cycler
- Thin-walled PCR tubes, 0.2 mL
- PyroMark pyrosequencing system (Qiagen)
- Gel electrophoresis equipment

***Reagents preparations***

*CT Conversion Reagent (EZ DNA Methylation-DirectTM Kit; D5020)*

*The CT Conversion Reagent provided with the EZ DNA Methylation-DirectTM Kit (D5020) is a solid mixture and must be prepared prior to first use. Please refer to the protocol provided with the kit for further details.*

1.  Add 790 μL of M-Solubilization Buffer to a tube of CT Conversion Reagent
2.  Add 300 μL of M-Dilution Buffer to the tube of CT Conversion Reagent
3.  Mix at room temperature with frequent vortexing or shaking for 10 min
4.  Add 160 μL of M-Reaction Buffer to the tube of CT Conversion Reagent
5.  Mix an additional 1 min

*M-Wash Buffer*

*The M-Wash Buffer provided with the EZ DNA Methylation-DirectTM Kit (D5020) must be prepared prior to first use. Please refer to the protocol provided with the kit for further details.*

1.  Add 24 mL of 100% ethanol to the 6 mL M-Wash Buffer concentrate (D5020)

***Procedure***

*DNA isolation (EZ DNA Methylation-DirectTM Kit; D5020)*

*Laser capture microdissected cells in pools of 50 should be collected in 8 µL TE buffer or PBS, as this is the optimal working volume for the EZ DNA Methylation-Direct Kit. In our case, cells were collected in 10 µL, hence volumes of the CT Conversion Reagent were adjusted accordingly to maintain the suggested reagent concentrations during the bisulfite conversion. For the original reagent volumes using laser capture microdissected cells in a total volume of 8 µL, please refer to the protocol provided with the kit. Further details on LDBSP sample handling can be found below.*

1.  Briefly centrifuge the tube with laser capture microdissected cells
2.  Add 11 µL M-Digestion Buffer (2X) to the sample
3.  Add 1 µL of Proteinase K to the sample
4.  Briefly vortex and centrifuge the tube
5.  Incubate the sample in a heating block at 50°C for overnight

*Bisulfite conversion of DNA (EZ DNA Methylation-DirectTM Kit; D5020)*

*When pipetting the sample, always use TipOne Low Retention Tips to minimize the loss of DNA due to handling. Prepared CT Conversion Reagent from the EZ DNA Methylation-Direct Kit should be divided into 143 µL ready-to-use aliquots in separate Eppendorf tubes, as this will allow one to use the same pipet tip per sample throughout the entire procedure. Similarly, the M-Binding Buffer was prepared in 600 µL ready-to-use aliquots per sample. Please find further details on handling below.*

1.  After incubation, transfer the digested sample to a PCR tube
2.  By using the same pipet tip, take 143 µL CT Conversion Reagent
3.  Use the CT Conversion Reagent to wash out the digestion tube
4.  Add the CT Conversion Reagent to the sample containing PCR tube
5.  Briefly vortex and centrifuge the tube
6.  Place the PCR tube in a thermal cycler and perform the following steps:
  - 98 °C for 8 min
  - 64 °C for 3.5 h
  - 4 °C storage
7.  Add 200 µL of M-Binding Buffer into a Zymo-Spin™ IC Column and place the column into a provided Collection Tube
8.  Pipet the bisulfite converted sample into the Zymo-Spin™ IC Column
9.  By using the same pipet tip, take 200 µL of M-Binding Buffer
10.  Use the M-Binding Buffer to wash out the PCR tube used for bisulfite conversion
11.  Add the 200 µL of M-Binding Buffer to the sample containing Zymo-Spin™ IC Column
12.  Repeat step 9–11 once more
13.  Close the cap and mix by inverting the column several times
14.  Centrifuge at full speed (>10,000× g) for 30 s
15.  Discard the flow-through
16.  Add 100 µL of M-Wash Buffer to the column
17.  Centrifuge at full speed (>10,000× g) for 30 s
18.  Add 200 µL of M-Desulphonation Buffer to the column and let stand at room temperature (20–30 °C) for 15–20 min
19.  Centrifuge at full speed (>10,000× g) for 30 s
20.  Add 200 µL of M-Wash Buffer to the column
21.  Centrifuge at full speed (>10,000× g) for 30 s
22.  Add 200 µL of M-Wash Buffer to the column
23.  Centrifuge at full speed (>10,000× g) for 30 s
24.  Place the column into a 1.5 mL LoBind Eppendorf tube
25.  Add 10 µL of M-Elution Buffer directly to the column matrix
26.  Centrifuge for 30 s at full speed (>10,000× g) to elute the DNA
27.  Add 10 µL of M-Elution Buffer directly to the column matrix
28.  Centrifuge for 30 s at full speed (>10,000× g) to elute the DNA

*NOTE: We strongly advise processing the samples directly for PCR amplification without intermediate storage by freezing to avoid potential loss of bisulfite-converted DNA due to freeze–thaw cycles.*

*Multiplex PCR*

*The volumes for the multiplex PCR can vary depending on the number of dilutions and target genes used for the LDBSP analysis. For illustrative purposes, we here provide an example based on 7 target genes and 22 dilutions, using a total volume of 25 µL for each individual PCR. Overall, the content of each individual PCR in relation to the buffer, dNTPs, and Taq DNA polymerase should be identical, whereas volumes of H_2_O vary depending on the number of target genes. Furthermore, always prepare several extra reactions for negative and positive controls, as well as for counteracting pipetting errors. The calculation demonstrated here is based on 22 dilutions (or reactions) for the samples, 2 control reactions, and 1 extra reaction for pipetting errors, giving a total of 25 reactions.*

1.  Prepare a single PCR mix containing the following reagents:
  - 2.5 μL PCR buffer (10X) with 20 mM MgCl_2_ per reaction
    → 25 × 2.5 μL= 62.5 μL
  - 0.5 μL 10 mM dNTP mix per reaction
    → 25 × 0.5 μL= 12.5 μL
  - 1 μL of the forward primer (10 μM stock) per gene per reaction
    → 25 × 7 genes × 1 μL = 175 μL
  - 1 μL of the reverse primer (10 μM stock) per gene per reaction
    → 25 × 7 genes × 1 μL = 175 μL
  - 0.2 μL (5 U/μL) FastStart™ Taq DNA Polymerase per reaction
    → 0.2 ∗ 25 = 5 μL
    → Components ‘a’-‘e’; total volume = 430 µL
2. Mix by shaking and then centrifuge the PCR mix
3. Divide the PCR mix into two separate Eppendorf tubes according to the necessary volumes
  - Tube 1, 22 reactions: 378.4 µL PCR mix
    → 430/25 × 22 = 378.4 µL
  - Tube 2, 2 reactions: 34.4 µL PCR mix
    → 430/25 × 2 = 34.4 µL
4. Add a required volume of H_2_O to both the PCR mixes (See details below)
  - Tube 1, 22 reactions: 151.6 µL H_2_O
    → 22 reactions × 25 µL total volume = 550 µL
    → Minus 378.4 µL PCR mix = 171.6 µL
    → Minus 20 µL bisulfite converted DNA = 151.6 µL
  - Tube 2, 2 reactions: 13.6 µL H_2_O
    → 2 reactions × 25 µL total volume = 50 µL
    → Minus 34.4 µL PCR mix = 15.6 µL
    → Minus 2 × 1 µL of template (Control DNA and/or H_2_O) = 13.6 µL
5. Mix by shaking and then centrifuge the PCR mixes
6. Add the complete PCR mix from ‘Tube 1′ to the 20 µL of bisulfite converted DNA
7. Mix thoroughly by pipetting up-and-down
8. By using the same pipet tip, dispense 25 µL of the mixture over 22 wells of a microtiter plate (mix thoroughly between dispensation steps)
9. Dispense 24 microliter of the PCR mix from ‘Tube 2′ into 2 wells of a microtiter plate
10. Add the necessary control templates to the latter 2 wells (e.g., positive and negative)
11. Seal the micro-titer plate
12. Place the micro-titer plate in a thermal cycler and perform the following steps:
  a. 95 °C for 5 min
  b. $\begin{array}{c} 95\;{}^{\circ}C\;\mathrm{for}\;30\;s\\ 56\;{}^{\circ}C\;\mathrm{for}\;30\;s\\ 72\;{}^{\circ}C\;\mathrm{for}\;30\;s \end{array}\}\begin{array}{c} \;\;\times 43\mathrm{cycles} \end{array}$
  c. 72 °C for 7 min
  d. 4 °C storage

*NOTE: The optimum primer concentrations, annealing temperature and required number of cycles should be tested a priori, both independently, as well as in multiplex formation. For the multiplex PCR amplifications, all primers need to be compatible with the same cycler conditions. The program displayed here was applied in the present study.*

*Singleplex PCR*

*Prepare a total volume of singleplex PCR mix that is sufficient for the number of the sample containing reactions (i.e., 22 in this case), negative controls, and some extra to counteract pipetting errors.*

1. Each PCR reaction should contain the following reagents per tube (Total volume of 25 μL):
  - 1 µL of Multiplex PCR product
  - 2.5 μL PCR buffer (10X) with 20 mM MgCl_2_
  - 0.5 μL 10 mM dNTP mix
  - 1 μL of the forward primer (10 μM stock)
  - 1 μL of the reverse primer (10 μM stock)
  - 0.2 μL (5 U/μL) FastStart™ Taq DNA Polymerase
  - 18.8 µL of H_2_O
2. Place the micro-titer plate in a thermal cycler and perform the following steps:
  a. 95 °C for 5 min
  b. $\begin{array}{c} 95\;{}^{\circ}C\;\mathrm{for}\;30\;s\\ 58\;{}^{\circ}C\;\mathrm{for}\;30\;s\\ 72\;{}^{\circ}C\;\mathrm{for}\;1\;\min \end{array}\}\begin{array}{c} \;\;\times 45\mathrm{cycles} \end{array}$
  c. 72 °C for 7 min
  d. 4 °C storage
3. Visualize the PCR products on a 2% agarose gel, using the Orange G DNA Loading Dye, the GelsGelStarTM Nucleic Acid Gel Stain, and a 100 bp ladder according to the manufacturer’s instructions.
4. Identify which of the reactions yield a gene-specific PCR product
5. Use the required amount of PCR product as suggested by the manufacturer’s instructions for subsequent pyrosequencing analysis.
6. Please refer to the manufacturer’s instructions for details on bisulfite pyrosequencing

*NOTE: The optimum primer concentrations, annealing temperature and required number of cycles should be tested a priori for each target gene, using both bisulfite converted DNA, as well as the multiplex product. The program displayed here was applied in the present study.*
