# Supplementary material for: Targeted Methylation Profiling of Single Laser-Capture Microdissected Post-Mortem Brain Cells by Adapted Limiting Dilution Bisulfite Pyrosequencing (LDBSP)
Source: Int J Mol Sci. 2022 Dec 8;23(24):15571. doi: 10.3390/ijms232415571 (PMC9779089; doi:10.3390/ijms232415571)

Table S1. Cohort demographics

	AD patients	Non-demented controls
N	12	12
Sex	Female	Female
Age of death; Mean (SD)	84.5 (5.04)	77.92 (13.00)
PMI; Mean (SD)	2.89 (0.96)	2.48 (0.62)
Plaque total; Mean (SD)	13.69 (1.71)	2.16 (3.54)
Tangle total; Mean (SD)	14.0 (1.36)	3.33 (2.38)
Braak stage; Range	V-VI	I – IV

Dorsal raphe nucleus (DRN) tissue was obtained from the Banner Sun Health Research Institute (BSHRI, Sun City, Arizona, US) as part of the Brain and Body Donation Program (BBDP). Displayed is the number of samples in each group, the sex of the subjects and the mean plus standard deviation (SD) of their age of death, post-mortem interval (PMI, in hours), total plaque load, and total tangle load, as well as their range of Braak stage.

Table S2. Primer overview

PCR primers

Gene	Forward primer (5'-3')	Reverse primer (5'-3')	Target region (GRCh37)	Product size (bp)
RHBDF2 (Multiplex)	AGGGTTTTGGGGATTTAGTG	CCAAAAACCTCACAAACAAATC	17:74475178-74475497	320
RHBDF2 (Singleplex)	AGGGTTTTGGGGATTTAGTG	(Bio-)CATCACCCCTTCCACACACTCAA	17:74475178-74475435	258
OXT (Multiplex)	GTTTAGGTTTTGTTAATGAAGAGGAA	ACCAAAACTAAAAACTCACCTTAC	20:3052117-3052440	324
OXT (Singleplex)	GTTTAGGTTTTGTTAATGAAGAGGAA	(Bio-)TCTTACCTCCCAAAAAACAATTC	20:3052117-3052392	276
TNXB (Multiplex)	TGTGAAGTAGGTTATTTAGGGGAAGA	CCTCCAAAACAAACCCTAACTAAAAACTCT	6:32063558-32063997	440
TNXB (Singleplex)	TGTGAAGTAGGTTATTTAGGGGAAGA	(Bio-)ACAAACCCTAACTAAAAACTCTTCCTACC	6:32063566-32063997	432
DNAJB13 (Multiplex)	GGTATTTTGGGAGGATGAGTTA	CCCTTCAAAAACAAACCAACTAAT	11:73668561-73668825	265
DNAJB13 (Singleplex)	GTATTTTGGGAGGATGAGTTATAATTG	(Bio-)CTTCAAAAACAAACCAACTAATAC	11:73668563-73668824	262
PGLYRP1 (Multiplex)	GTTGGGGAGAGTTTAGGTAAG	ATTCCTATTAAATTATTCAATATTCCACT	19:46526238-46526634	397
PGLYRP1 (Singleplex)	TGGGGAGAGTTTAGGTAAGTA	(Bio-)TCCTATTAAATTATTCAATATTCCACTAA	19:46526240-46526632	393
C3 (Multiplex)	GATTGGGTTTTATTTGAGTGTAAGAT	CCAACAATAAATTAAAAACTCCAATCTTC	19:6713187-6713627	441

C3 (Singleplex)	GATTGGGTTTTATTTGAGTGTAAGAT	(Bio-)AACCTAAAACCCTCCTTATCTAT	19:6713187-6713550	364
LMX1B (Multiplex)	TTTTTTAAGGGGGTGGAGTAGAG	CCTCACCTCCCCAAACTTCCTAACATT	9:129375928-129376184	257
LMX1B (Singleplex)	GGGGGTGGAGTAGAGGGA	(Bio-)CCTCACCTCCCCAAACTTCCTAACATT	9:129375936-129376184	249

Pyrosequencing primers

Gene	Sequencing primer (5'-3')	Number of CpGs	PyroMark Orientation	Target region (GRCh37)	Primer
RHBDF2	TTTTTGGTTGGGGAG	5	Upper strand(5'-3')	17:74475230-74475271	1
RHBDF2	GTATGTTGTAGTGGTG	5	Upper strand(5'-3')	17:74475287-74475332	2
RHBDF2	GATTTTAAATTATTGGGTTG	4	Upper strand(5'-3')	17:74475355-74475403	3
OXT	GGTTTTGTTAATGAAGAGGAA	5	Upper strand (5'-3')	20:3052143-3052173	1
OXT	ATATTATTAATTTTTTAAATAGAG	2	Upper strand (5'-3')	20:3052215-3052224	2
OXT	AATGTTTAGGTATAAAAAGGT	5	Upper strand (5'-3')	20:3052248-3052297	3
TNXB	GGTTAGTGTTTAGATGGG	5	Lower strand (5'-3')	6:32063874-32063913	1
TNXB	AGGATTGAGGTGTGA	4	Lower strand (5'-3')	6:32063823-32063867	2
TNXB	GGTGTGTGTATTTGTTG	2	Lower strand (5'-3')	6:32063774-32063806	3
DNAJB13	GGATGAGTTATAATTGGG	5	Lower strand (5'-3')	11:73668745-73668795	1
DNAJB13	TTTTGTTATTTGTTTGAAAA	4	Lower strand (5'-3')	11:73668618-73668673	2
PGLYRP1	TTTTTTTTTGGTTGGGTTAG	1	Upper strand (5'-3')	19:46526451-46526454	1
PGLYRP1	GAGGGATGTATTGTGG	3	Upper strand (5'-3')	19:46526468-46526509	2
PGLYRP1	GTTTAGTAGGGAGGG	5	Upper strand (5'-3')	19:46526524-46526555	3
C3	TTTTAATTTGAGAAGGGAGA	2	Upper strand (5'-3')	19:6713340-6713376	1
C3	TGGGGTAGGGATTAAA	4	Upper strand (5'-3')	19:6713398-6713426	2
C3	ATTTAGGTAGGGAAA	2	Upper strand (5'-3')	19:6713450-6713475	3
LMX1B	GTTTAGAAGAAGATTAAAATTTTTG	7	Upper strand (5'-3')	9:129375982-129376016	1

Displayed is an overview of the polymerase chain reaction (PCR) and pyrosequencing primers that were used in this study. Provided for each target gene are the multiplex and singleplex primer sequences (Bio, biotinylated), as well as corresponding genomic coordinates (Ensembl GRCh37 assembly) and amplicon sizes in base pairs

(bp). For the pyrosequencing primers, the total number of CpG sites, the targeted region for sequencing, and the orientation of the assay in the PyroMark assay design software 2.0 are listed per gene.

Figure S1. Allele overview. Displayed for each target gene are the recovered alleles and their respective methylation patterns. Each horizontal row represents a single product-yielding polymerase chain reaction (PCR) containing either one, two or three alleles, indicated with 1, 2 or 3, respectively. Cytosine-phosphate-guanine (CpG) sites are depicted as boxes and colored according to their methylation pattern. Please refer to the figure legend for further details on color-coding.

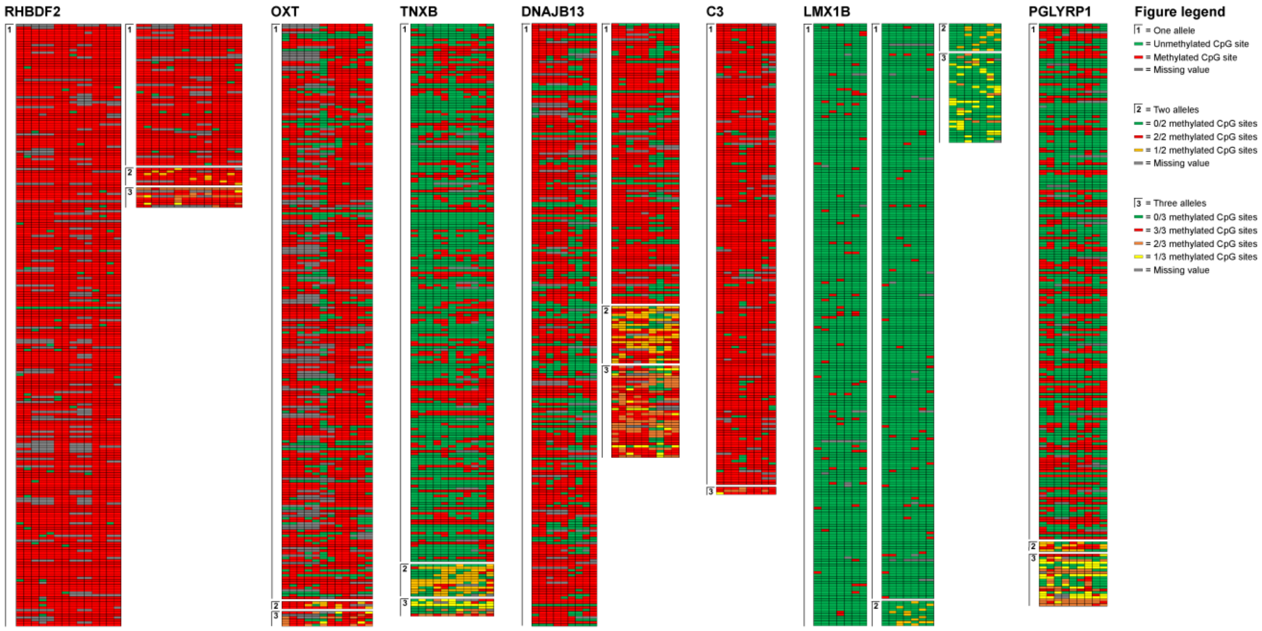

Supplement: Supplementary file 1 [file ijms-23-15571-s001.zip › ijms-2028915-supplementary.pdf]
